# Reconstruction of the medial patellofemoral ligament using two blind transverse semi-patella tunnels and an implant-free technique for patellar fixation: a technical note

**DOI:** 10.1186/s13018-020-02161-z

**Published:** 2021-01-07

**Authors:** Vasileios Raoulis, Aristeidis Zibis, Apostolos Fyllos, Michael-Alexander Malahias, Konstantinos Banios, Michael Hantes

**Affiliations:** 1 Department of Orthopaedic Surgery, University Hospital of Larissa, 43, LamprouKatsonis str, 41221 Larissa, Greece; 2 Department of Anatomy, University Hospital of Larissa, 43, LamprouKatsonis str, 41221 Larissa, Greece; 3 Complex Joint Reconstruction Center, Hospital for Special Surgery, 535 East 72nd Street, New York, NY 10021 USA

**Keywords:** MPFL reconstruction, Double-bundle MPFL technique, Patella instability, Implant-free patella fixation

## Abstract

**Background:**

The double-bundle technique with two points of patellar fixation in the upper half of the patella replicating the broad attachment site of the native medial patellofemoral ligament (MPFL) is the most commonly performed procedure for MPFL reconstruction. Complete transverse patella tunnels pose a threat to the integrity of the patella. We present an implant-free, double-bundle technique for MPFL reconstruction with gracilis autograft, overcoming the problem of complete patella bone tunnels and over-drilling.

**Methods:**

After standard gracilis graft harvesting, the anteromedial side of the patella is exposed. With the guidance of an anterior-cruciate-ligament (ACL) tibia-aiming device, two 2-mm parallel guide pins are inserted from medial to lateral at the upper half of the patella. The two guide pins are over-drilled with a cannulated 4.5-mm drill bit 2-cm deep, to create two transverse blind semi-patellar tunnels. For the femoral fixation, a 2.4-mm guide pin with an eyelet is drilled at the Schöttle point and over-reamed with a 6-mm cannulated reamer to a depth of 30 mm. The two free ends of the graft (with two running Krakow sutures placed) are pulled into the two patella tunnels and the graft sutures are tied together with tension for stable graft fixation at the lateral patella rim. With the help of a femoral suture loop (which is inserted in the femoral bone tunnel), the graft-loop is advanced into the femoral bone tunnel and the graft is finally fixed with a 7-mm interference screw at 30° of knee flexion.

**Results:**

The utilization of blind transverse tunnels (not trans-patellar tunnels) offers the advantage of avoiding stress risers at the patella. Thanks to the ACL tibia aiming device, multiple drilling, and breaching of the anterior patellar cortex or articular surface of the patella is avoided.

**Conclusions:**

This implant-free, and consequently affordable technique, isolated or combined with bony procedures, minimizes possibilities for perioperative bony complications at the patella fixation site.

## Background

Μedial patellofemoral ligament (MPFL) reconstruction is currently the first-choice soft-tissue procedure for patients requiring surgery after more than 1 or 2 episodes of patellar dislocation [1–3]. An isolated reconstruction of the MPFL or combined with other bone procedures such as tibial tubercle osteotomy or trochleoplasty could be performed, depending on patient’s anatomy. A variety of surgical techniques for anatomic reconstruction of the MPFL are available in the literature; however, there is no consensus as to which technique yields the best clinical outcome [4–15]. A two-bundle technique, with free tendon grafts and two points of patellar fixation (upper half of the patella), replicates adequately the broad attachment site of the native MPFL (fan-shaped insertion of the MPFL) on the patella [11, 14, 15]. Regarding the graft choice, the majority of surgeons are using hamstring tendons as the graft of choice [2, 5, 8–13, 16–19]. The differences of these surgical techniques concern patella fixation, since femoral fixation with a bio-composite screw at the Schöttle point allows isometric adjustments of the graft, resulting in a good clinical outcome and it is generally accepted [11–13]. Some of the popular techniques include utilization of implants such as suture anchors [4, 7, 9] and interference screws for graft fixation of the patella [11, 17]. Others describe anatomic hardware-free patellar fixation, where the graft is passed through 2 bone tunnels in the patella or a bone bridge is created on the medial margin of the patella [4–8]. Complete reaming that creates transverse complete bone tunnels that pass completely through the patella hold the risk of causing patellar fractures, as they act like stress-risers [20–23].

We present a double-bundle technique for MPFL reconstruction, without patella implants, overcoming the problem of complete patella bone tunnels and overdrilling.

## Surgical technique

**Fig. 1:**
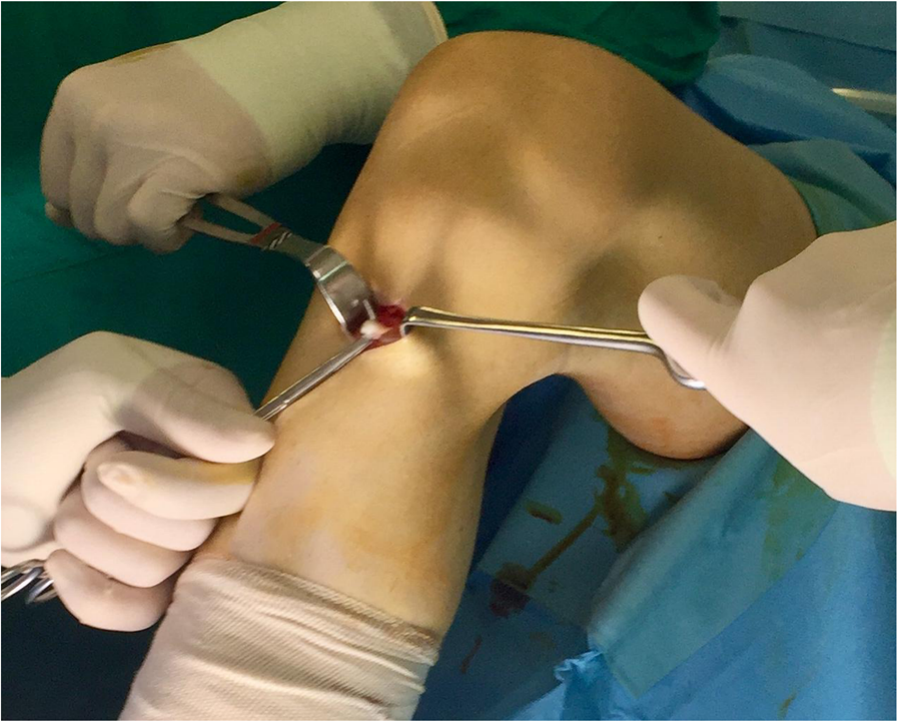
Identifying the gracilis autograft before harvesting

The patient is placed in a supine position, and a tourniquet is applied to the upper thigh. Arthroscopy would be warranted prior to MPFL reconstruction, in case of suspected intra-articular pathology (loose bodies, cartilage lesions of the patellofemoral joint). Gracilis tendon autograft is harvested through a vertical or horizontal incision which is placed 2 cm medially to the pes anserinus. The overlying sartorial fascia and the pes anserinus bursa are exposed and incised (Fig. 1). Adhesions are excised, muscular attachments are released, and the tendon is delivered with a tendon stripper. After the preparation of the gracilis tendon graft (approximately 20–21 cm), a running locking Krackow suture is placed up to approximately 2 cm from each free end with a no. 2 non-absorbable suture (Ethibond suture 2).

A solution of 100 ml of sterile saline is prepared in a tray and mixed with 500 mg of vancomycin powder. The prepared graft is immersed in the tray and is then wrapped in gauze that has been saturated with the vancomycin solution beforehand to eliminate graft contamination. The graft is kept there until the implantation.

**Fig. 2:**
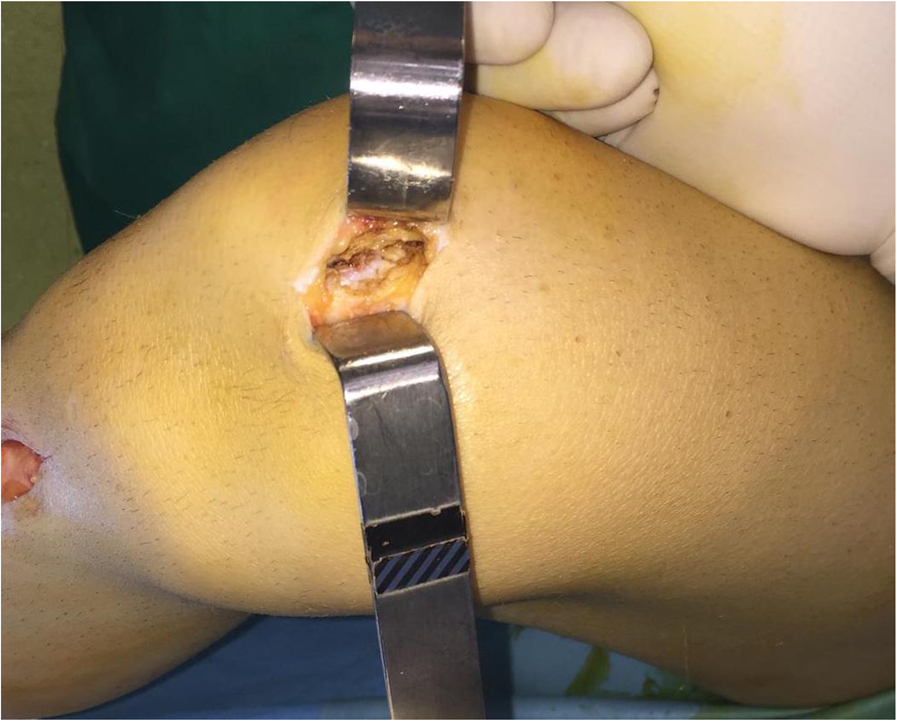
Anteromedial side patella exposure before tunnel-drilling

**Fig. 3:**
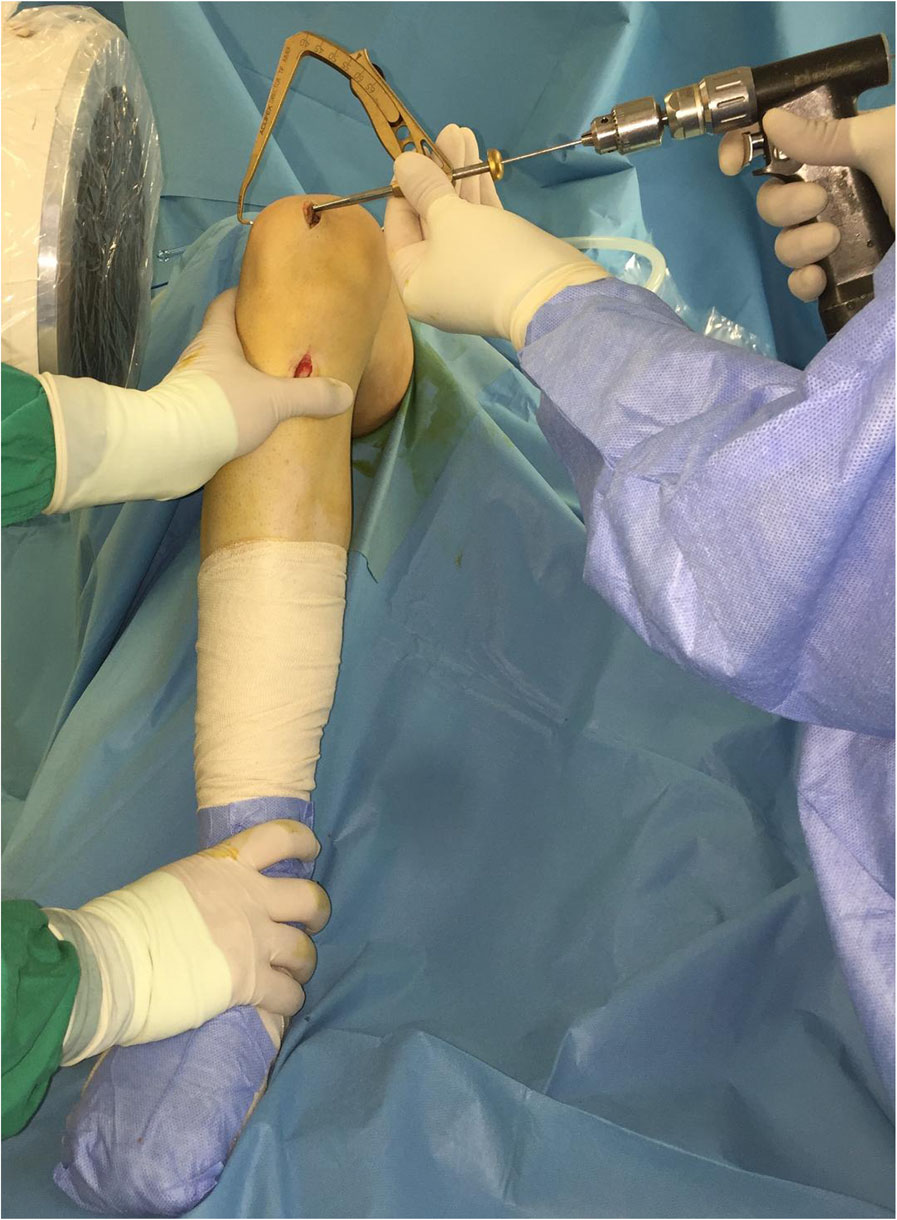
With the help of an anterior-cruciate-ligament-reconstruction (ACL) tibial-aiming device and under fluoroscopy a 2-mm guide pin with an eyelet is drilled from medial to lateral at the patella

With the knee flexed at 90°, a longitudinal incision (2–3 cm) is performed on the anteromedial side of the patella and the medial aspect of the patella is exposed all the way to the bone surface by electrocautery, without penetrating the capsule (Fig. 2). A guide pin of 2.0-mm diameter with an eyelet is transversely inserted from the midpoint of the medial edge of the patella to the lateral border, with the help of an anterior-cruciate-ligament-reconstruction (ACL) tibial-aiming device to avoid breaching either the articular surface or the anterior cortex (Fig. 3). For the correct placement of the ACL tibial-aiming device spike on the upper and lateral border of the patella, sometimes a small lateral incision (1 cm) might be necessary.

**Fig. 4:**
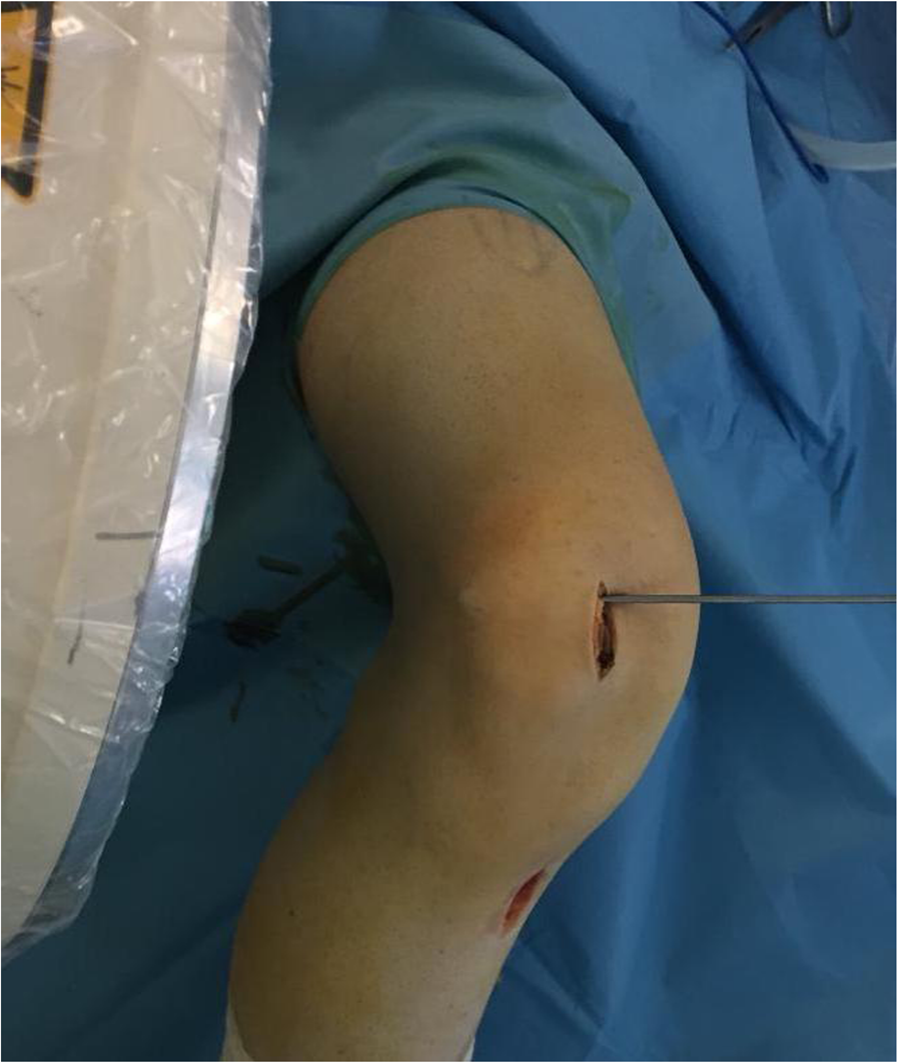
The direction of the guide pin is transverse, perpendicular to the longitudinal axis of the patella and parallel to the coronal patella plane

**Fig. 5:**
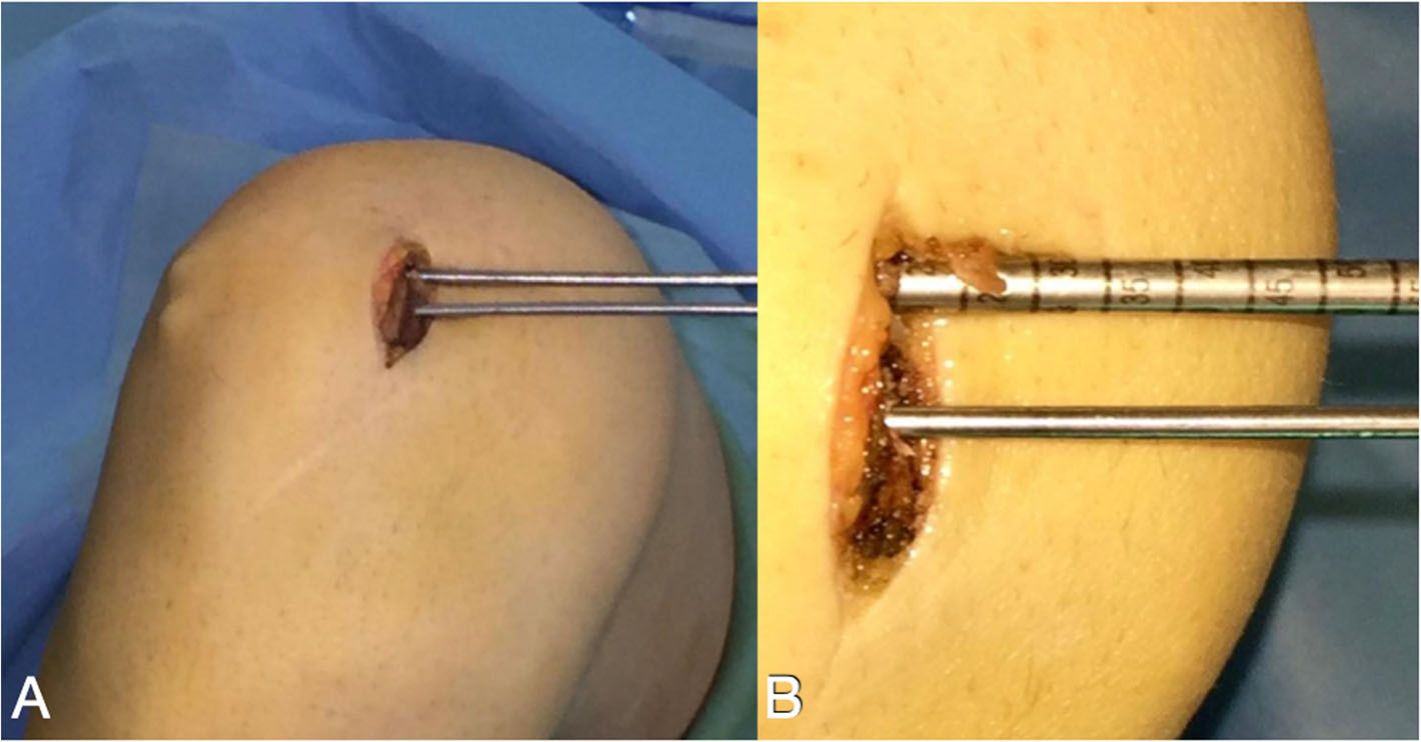
**a** The second guide pin is inserted 15 mm proximally and transversely to the first pin. **b** Both guide pins are over-drilled with a cannulated 4.5-mm drill bit 2-cm deep

**Fig. 6:**
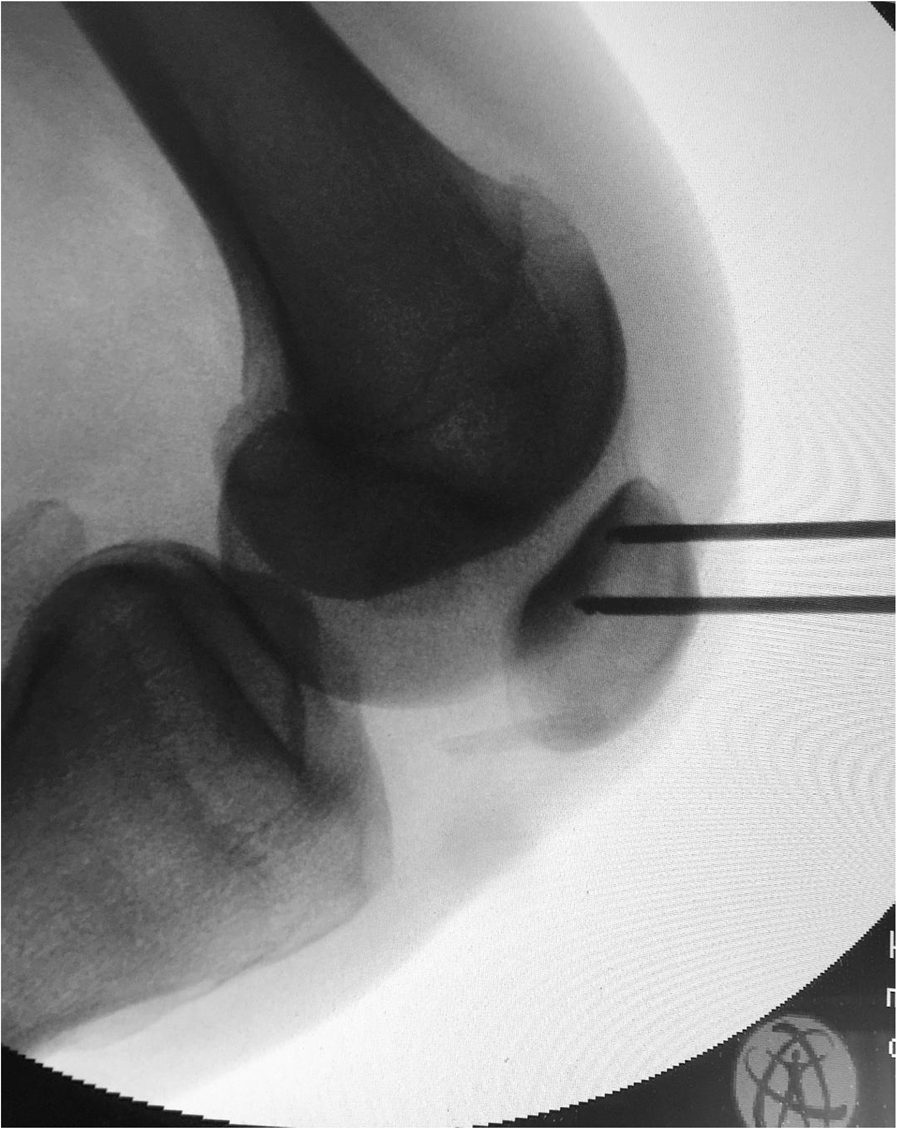
Confirmation by fluoroscopy of the appropriate placement of the guide pins

**Fig. 7:**
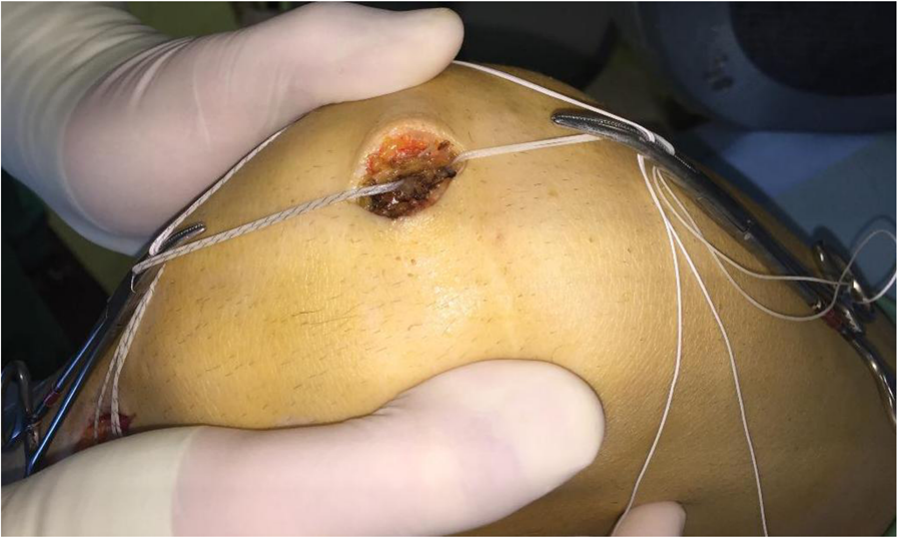
Two suture loops are inserted into the tunnels, with the help of the guide pin with the eyelet

The direction guide pin is drilled in a transverse fashion, perpendicular to the longitudinal axis of the patella and parallel to the coronal patella plane (Fig. 4). The appropriate placement of the guide pin is confirmed by fluoroscopy. A second guide pin is placed 15 mm proximally and parallel to the first pin, as checked using a ruler and the two guide pins are over-drilled with a cannulated 4.5-mm drill bit 2-cm deep, to create two 2-cm transverse bone tunnels at the medial side of the patella (Fig. 5). The appropriate placement of the second guide pin is also confirmed by fluoroscopy (Fig. 6). Two suture loops are inserted into the tunnels, with the loop lying on the medial side (Fig. 7). Considering the small size and the special shape of the patella, it is very important to drill two parallel transverse semi-patellar tunnels with the first attempt so that we do not end up with a “Swiss cheese” patella. This is a very important step of the operation (ACL tibia device) that differentiates it from other surgical techniques described in the literature before.

**Fig. 8:**
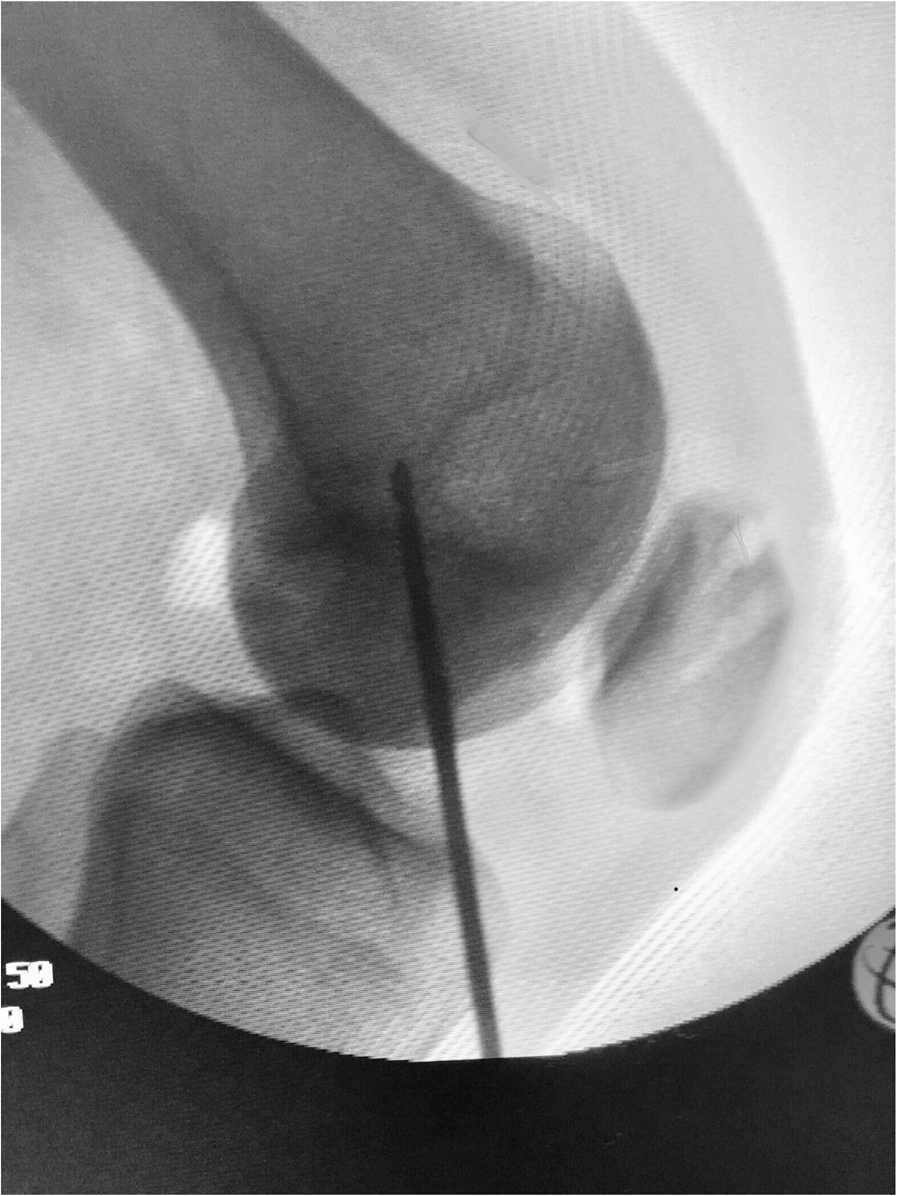
Under fluoroscopy a 2-mm guide pin with an eyelet is drilled at the Schöttle point

**Fig. 9:**
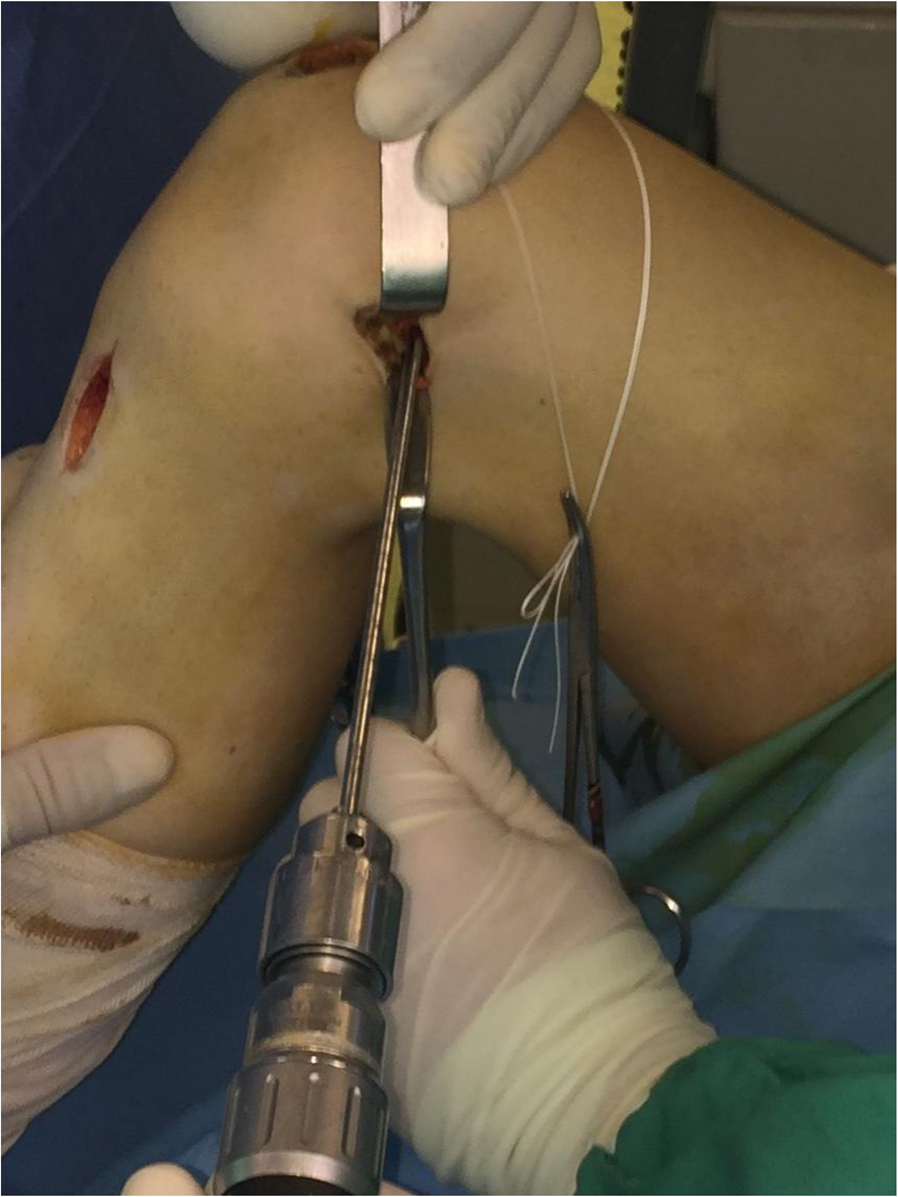
A 6-mm cannulated reamer is introduced over the guide pin to a depth of 30 mm

**Fig. 10:**
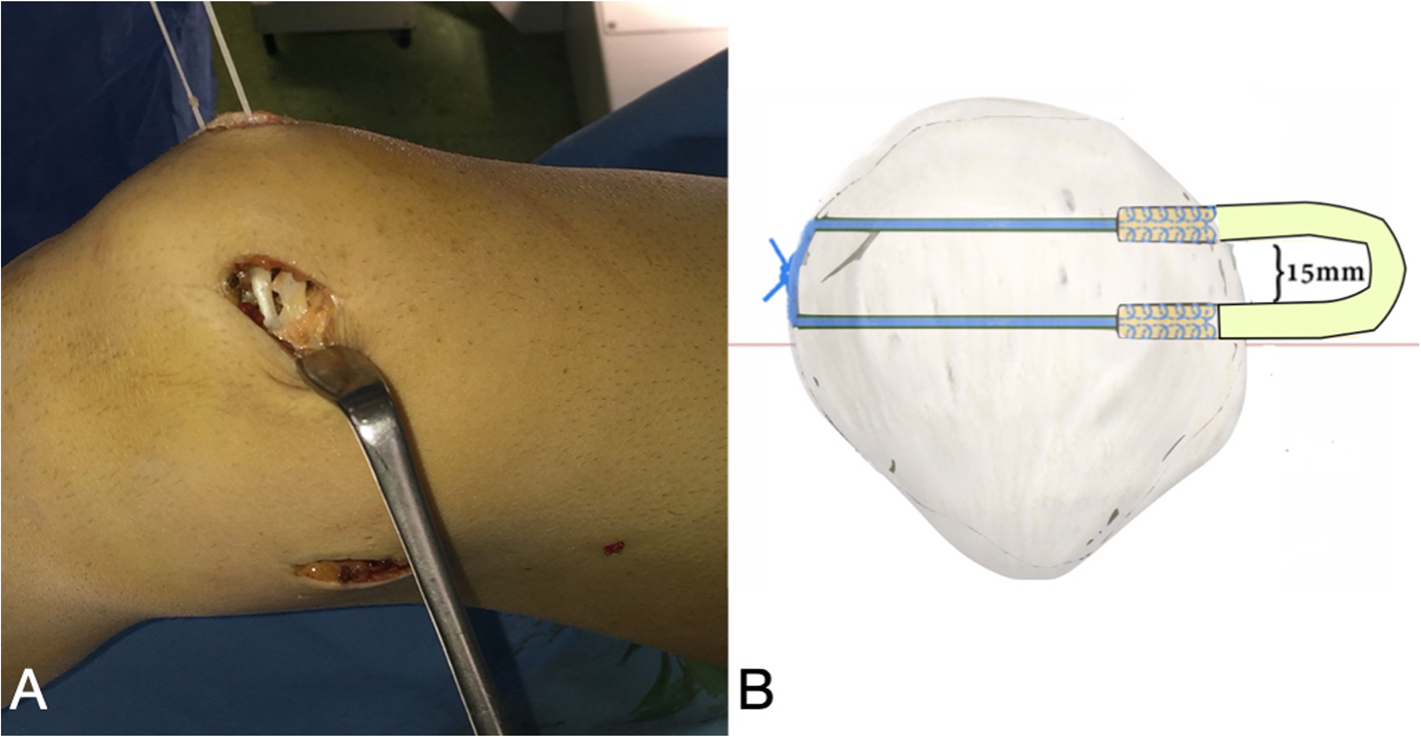
**a** The free ends of the tendon graft are pulled into the 2 patella tunnels. **b** Drawing of the patella fixation utilizing two transverse blind semi-patellar tunnels without hardware by suture knot at the lateral aspect of the patella

The knee is then flexed to 30°, and the adductor tubercle is identified by palpation and under fluoroscopic guidance, a 2.4-mm guide pin with an eyelet is drilled at the Schöttle point (Fig. 8). A 2-cm skin incision is made over the guide pin at the adductor tubercle (retractors can be used for better visualization). Afterwards the guide pin is over-reamed with a 6-mm cannulated reamer to a depth of 30 mm (Fig. 9). A non-absorbable suture loop is passed through the eyelet, and the guide pin is pulled out from the lateral aspect of the femur so that the suture-loop stays on the medial side of the femoral tunnel. Before positioning the graft, the second and the third layer of the medial patellofemoral complex are separated by dissection down to the femoral insertion side, while care is taken to leave the capsule intact. The previously prepared graft is then brought to the front table, passed through the patellar incision, so that the sutures of each free graft-end are passed through the suture-loops at the patella tunnels and then pulled out from medial to lateral. Both ends of the tendon graft are pulled into the 2 patella tunnels, and the graft sutures are tied together with tension for stable graft fixation at the lateral patella rim. If a small lateral incision has been previously made for the ACL tibia guide, then the suture ends are passed through that incision and the knot is tied and buried (Fig. 10).

**Fig. 11:**
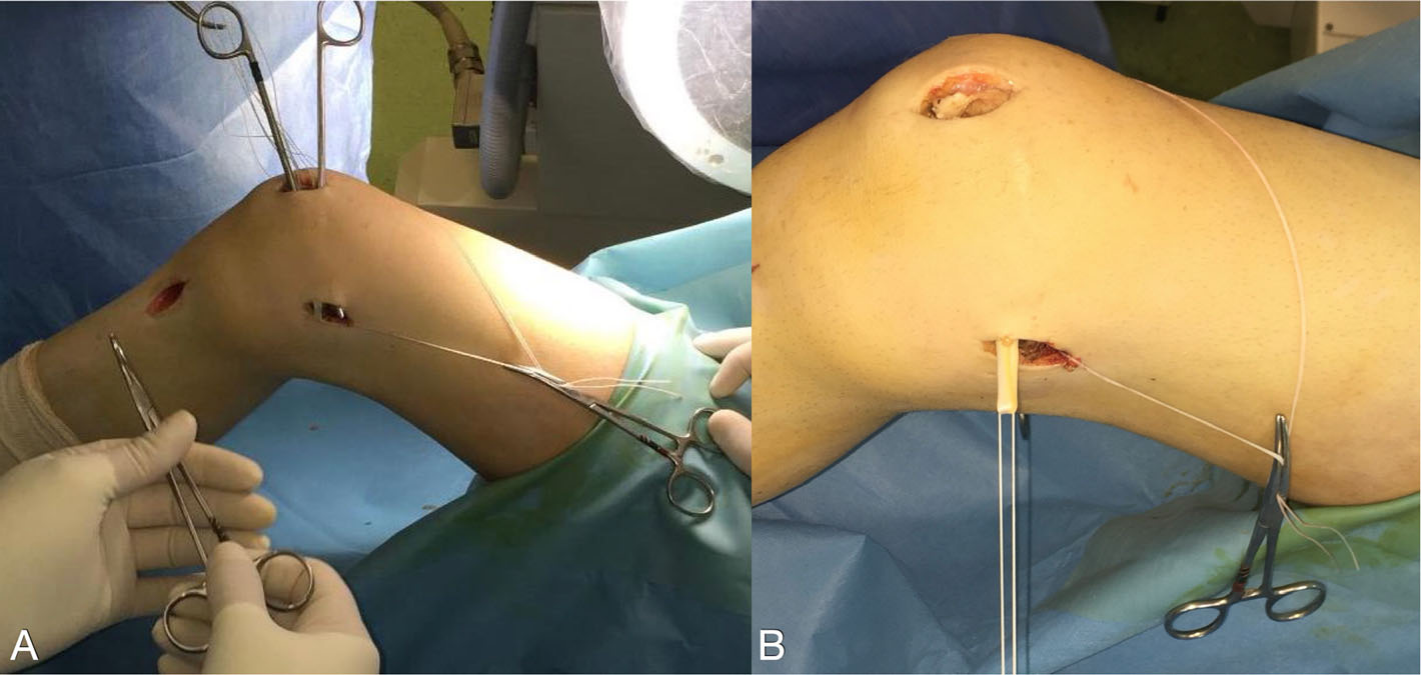
**a**, **b** The graft loop is pulled by the suture downwards between the second and the third layer of the medial patellofemoral complex to the created femoral tunnel

**Fig. 12:**
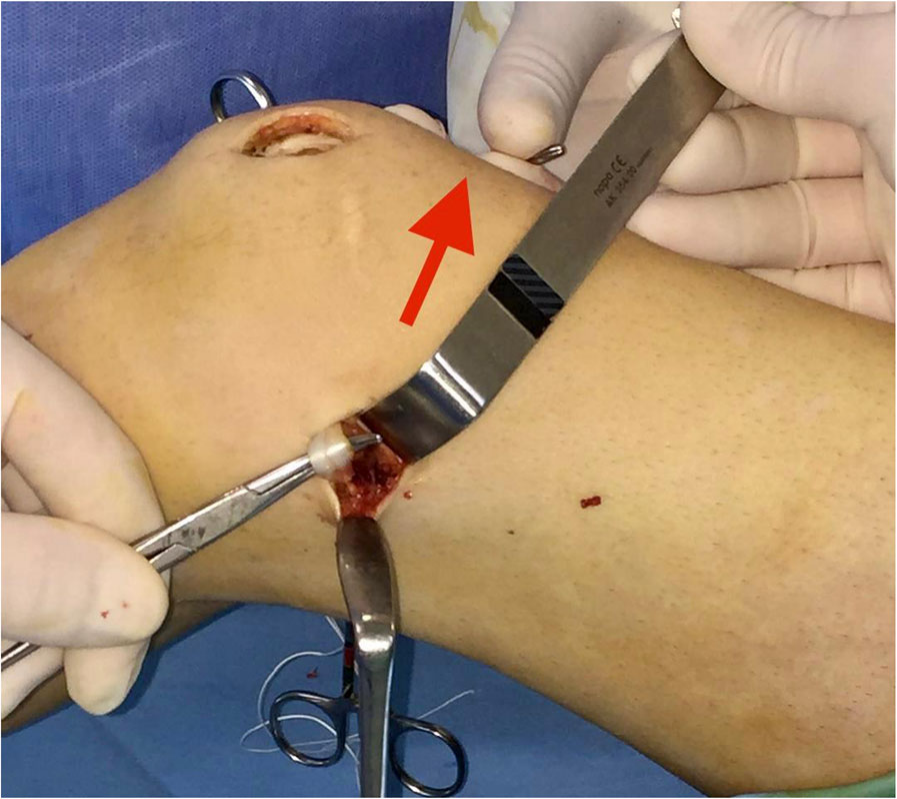
Utilizing a mosquito-clamp as fulcrum to soften the steep angle, while the assistant is pulling the suture-loop under light tension (red arrow)

A non-absorbable suture no. 2 is passed around in the center of the gracilis tendon loop is retrieved in a retrograde fashion between the second and third capsular layer to the femoral insertion of the MPFL. The graft loop is pulled by the suture downwards to the created femoral tunnel (Fig. 11). With the help of a femoral suture loop (which is inserted in the femoral bone tunnel), the graft-loop is finally advanced into the femoral bone tunnel for 2 cm or more, using a mosquito-clamp as a fulcrum to soften the steep angle (Fig. 12).

**Fig. 13:**
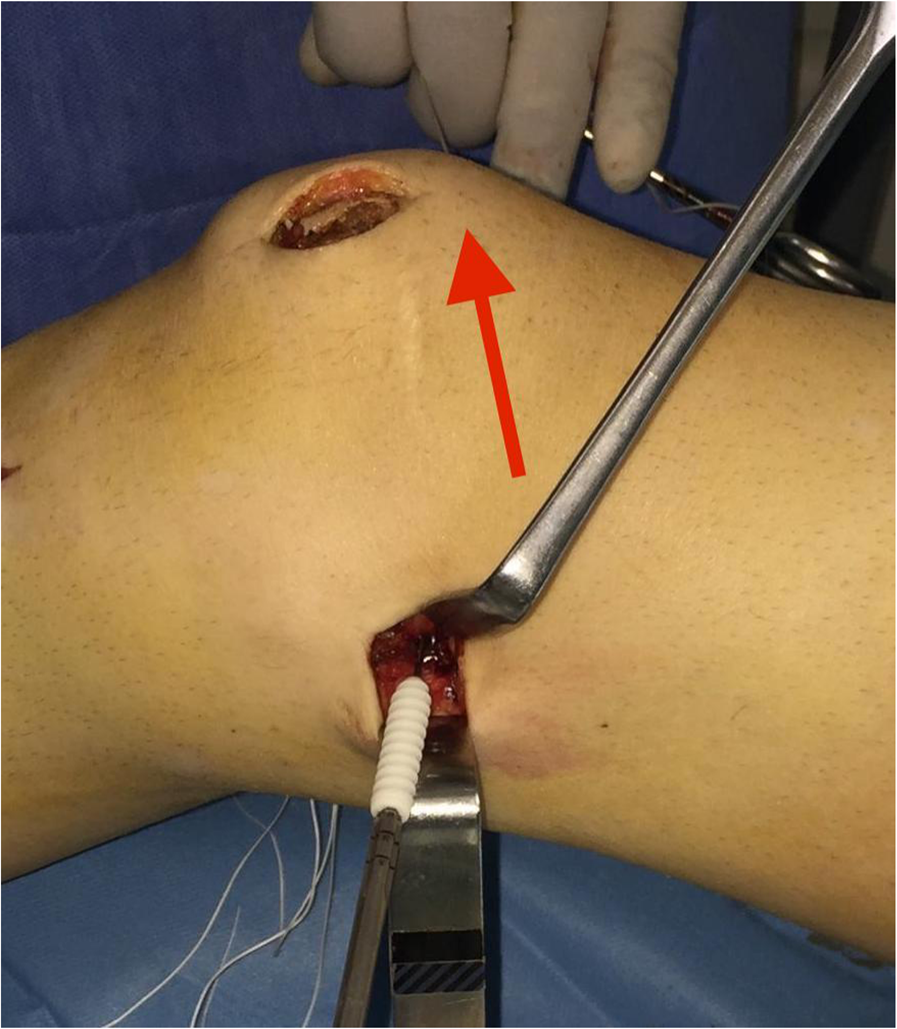
Graft fixation with a 7-mm interference screw at 30° of knee flexion, while the assistant is pulling the suture-loop under moderate tension (red arrow)

The knee is cycled 20 times with moderate tension on the graft and finally fixed with a 7-mm interference screw at 20–30° of knee flexion (Fig. 13). Attention is paid not to over-tighten the graft. Usually, the tension is adjusted so that the patella can be displaced laterally only 30 to 40% of its maximum transverse diameter.

## Rehabilitation

Postoperatively, the knee is kept in a brace for 4 weeks while allowing weight-bearing as tolerated. The brace is initially locked in extension for 1 week, and then, flexion is gradually permitted so that after 4 weeks the patient has 90° flexion. Quadricep exercises (isometric) are encouraged from the first week. Full range of motion is allowed after 6 weeks. Light running is initiated after 8 weeks. Patients can return to contact sports at 9 months after surgery.

## Discussion

The use of screws and anchors in the patella fixation is less time-consuming and easier to use than hardware free-fixation techniques, but it has been accused of causing pain and irritation at the insertion side [20]. On the other hand, patellar fixation techniques without implants have the advantage of being less costly. Complete reaming and complete transverse bone tunnels might cause patellar fractures, or bone bridge collapse, as they act like stress-risers [20–23]. The technique described in this technical note has the advantage of avoiding breaching the anterior cortex of the patella, minimizes the bone tunnels, utilizes blind transverse tunnels (not trans-patellar tunnels), and avoids devascularization of the superior pole of the patella thanks to small incision and minimal exposure. In addition, by introducing the use of the ACL aiming-tibia device for the creation of patella tunnels, the damage of patella integrity and strength is significantly reduced. However, since the final clinical outcome characterizes the procedure, the treating surgeon should be vigilant during the rehabilitation protocol, and in close communication with the patient, since problems can occur on every step of the way. A common problem is persistent postoperative stiffness that can occur if the patient is not able to follow the established rehabilitation protocol.

A useful tip one should bear in mind when choosing this technique is to ensure graft length of at least 20 cm, since the anatomical length of the native MPFL has an average length of 5.3 cm [10] and more working length is required for femoral and patellar fixation. The mean length of the gracilis tendon is reported at 27.7 cm, of which 20 cm are prepared and the graft is folded in half [24]. From the folded graft 2 cm from the free ends of the gracilis enters in the patella tunnels, there are 3 cm left for the femur and 5 cm for the reconstructed MPFL. When inserting the femoral screw, as the goal is not to over-tension the reconstructed MPFL, the 3 cm tunnel in the Schottle point is considered to be sufficient. Αutologous gracilis tendon graft has been proven clinically and biomechanically to be suitable for MPFL reconstruction [6, 12]. A common perioperative complication when harvesting the graft is injury the infrapatellar branch of the saphenous nerve, which usually lies in the subcutaneous fat at the point of the incision. Care should also be taken to prevent any injury to the underlying superficial medial collateral ligament. Graft embalmment in vancomycin solution prior to final placement has yielded excellent results in terms of infection prevention [25, 26]. Furthermore, the surgeon should avoid inserting the graft distally to the native insertion of the MPFL to avoid constraint of the distal patellar pole. To that end, two convergent holes should be drilled in the proximal half of the patella. During preparation of the 2 patellar tunnels, or during passage of an oversized tendon graft through a tight patellar tunnel, the bone bridge overlying the patellar tunnel may collapse. Therefore, a cortical bone bridge of at least 15 mm should be left between them to avoid fracture or bone bridge collapse. This prerequisite however creates a technical issue for patients with small-sized patella (pediatric or Asian population) and preferably should be avoided in this kind of patients. The mean patella length has been found to range from 31.3 to 42.04 mm between genders and in various ethnicities [27–29]. One should not forget to mention that over-tightening of the graft leads to elevated medial patellofemoral forces, resulting in an overconstrained patella that is painful, and could lead to patellofemoral osteoarthritis.

We have been using this technique in our department for several years, isolated or combined with bony procedures, without perioperative or postoperative complications and with good clinical results. This is a useful modification of an established technique [10, 30], which minimizes possibilities for perioperative bony complications at the patella fixation, and we introduce the utilization of ACL tibia aiming device.

## Data Availability

Not applicable.

## References

[1] Erickson BJ, Nguyen J, Gasik K, Gruber S, Brady J, Shubin Stein BE (2019). Isolated medial patellofemoral ligament reconstruction for patellar instability regardless of tibial tubercle-trochlear groove distance and patellar height: outcomes at 1 and 2 years. Am J Sports Med.

[2] Hiemstra LA, Kerslake SA, Lafave MR (2019). Influence of risky pathoanatomy and demographic factors on clinical outcomes after isolated medial patellofemoral ligament reconstruction: a regression analysis. Am J Sports Med.

[3] Mulliez A, Lambrecht D, Verbruggen D, Van Der Straeten C, Verdonk P, Victor J (2017). Clinical outcome in MPFL reconstruction with and without tuberositas transposition. Knee Surg Sports Traumatol Arthrosc.

[4] Hapa O, Akşahin E, Özden R (2012). Aperture fixation instead of transverse tunnels at the patella for medial patellofemoral ligament reconstruction. Knee Surg Sports Traumatol Arthrosc.

[5] Hinterwimmer S, Imhoff AB, Minzlaff P, Saier T, Rosenstiel N, Hawe W (2013). Anatomical two-bundle medial patellofemoral ligament reconstruction with hardware-free patellar graft fixation: technical note and preliminary results. Knee Surg Sports Traumatol Arthrosc.

[6] Kyung H-S, Kim H-J (2015). Medial patellofemoral ligament reconstruction: a comprehensive review. Knee Surg Relat Res.

[7] Lenschow S, Schliemann B, Gestring J, Herbort M, Schulze M, Kosters C (2013). Medial patellofemoral ligament reconstruction: fixation strength of 5 different techniques for graft fixation at the patella. Arthroscopy.

[8] Panni AS, Alam M, Cerciello S, Vasso M, Maffulli N (2011). Medial patellofemoral ligament reconstruction with a divergent patellar transverse 2- tunnel technique. Am J Sports Med.

[9] Russ SD, Tompkins M, Nuckley D, Macalena J (2015). Biomechanical comparison of patellar fixation techniques in medial patellofemoral ligament reconstruction. Am J Sports Med.

[10] Siebold R, Borbon CAV (2012). Arthroscopic extraarticular reconstruction of the medial patellofemoral ligament with gracilis tendon autograft - surgical technique. Knee Surg Sports Traumatol Arthrosc.

[11] Schöttle PB, Hensler D, Imhoff AB (2010). Anatomical double-bundle MPFL reconstruction with an aperture fixation. Knee Surg Sports Traumatol Arthrosc.

[12] Schöttle PB, Schmeling A, Romero J, Weiler A (2009). Anatomical reconstruction of the medial patellofemoral ligament using a free gracilis autograft. Arch Orthop Trauma Surg.

[13] Schöttle PB, Fucentese SF, Romero J (2005). Clinical and radiological outcome of medial patellofemoral ligament reconstruction with a semitendinosus autograft for patella instability. Knee Surg Sports Traumatol Arthrosc.

[14] Wang C-H, Ma L-F, Zhou J-W, Ji G, Wang HY, Wang F (2013). Double-bundle anatomical versus single-bundle isometric medial patellofemoral ligament reconstruction for patellar dislocation. Int Orthop.

[15] Wang Q, Huang W, Cai D, Huang H (2017). Biomechanical comparison of single- and double-bundle medial patellofemoral ligament reconstruction. J Orthop Surg Res.

[16] Deie M, Ochi M, Sumen Y, Adachi N, Kobayashi K, Yasumoto M (2005). A long-term follow-up study after medial patellofemoral ligament reconstruction using the transferred semitendinosus tendon for patellar dislocation. Knee Surg Sports Traumatol Arthrosc.

[17] Panagopoulos A, van Niekerk L, Triantafillopoulos IK (2008). MPFL reconstruction for recurrent patellar dislocation: a new surgical technique and review of the literature. Int J Sports Med.

[18] Russo F, Doan J, Chase DC, Farnsworth CL, Pennock AT (2016). Medial patellofemoral ligament reconstruction: fixation technique biomechanics. J Knee Surg.

[19] Saper MG, Meijer K, Winnier S, Popovich JJR, Andrews JR, Roth C (2017). Biomechanical evaluation of classic solid and all-soft suture anchors for medial patellofemoral ligament reconstruction. Am J Sports Med.

[20] Shah JN, Howard JS, Flanigan DC, Brophy RH, Carey JL, Lattermann C (2012). A systematic review of complications and failures associated with medial patellofemoral ligament reconstruction for recurrent patellar dislocation. Am J Sports Med.

[21] Parikh SN, Wall EJ (2011). Patellar fracture after medial patellofemoral ligamentsurgery: a report of five cases. J Bone Joint Surg Am.

[22] Parikh SN, Lykissas MG, Gkiatas I (2018). Predicting risk of recurrent patellar dislocation. Curr Rev Musculoskelet Med.

[23] Tompkins M, Arendt EA (2012). Complications in patellofemoral surgery. Sports Med Arthrosc.

[24] Janssen RPA, van der Velden MJF, van den Besselaar M, Reijman M (2017). Prediction of length and diameter of hamstring tendon autografts for knee ligament surgery in Caucasians. Knee Surg Sports Traumatol Arthrosc.

[25] Schuster P, Schlumberger M, Mayer P, et al. Lower incidence of post-operative septic arthritis following revision anterior cruciate ligament reconstruction with quadriceps tendon compared to hamstring tendons [published online ahead of print, 2020 Feb 4]. Knee Surg Sports Traumatol Arthrosc. 2020. 10.1007/s00167-020-05878-w. 10.1007/s00167-020-05878-w 32020252

[26] Banios K, Komnos GA, Raoulis V, Bareka M, Chalatsis G, Hantes ME. Soaking of autografts with vancomycin is highly effective on preventing postoperative septic arthritis in patients undergoing ACL reconstruction with hamstrings autografts [published online ahead of print, 2020 May 3]. Knee Surg Sports Traumatol Arthrosc. 2020. 10.1007/s00167-020-06040-2. 10.1007/s00167-020-06040-2 32363476

[27] Huang AB, Luo X, Song CH, Zhang JY, Yang YQ, Yu JK (2015). Comprehensive assessment of patellar morphology using computed tomography-based three-dimensional computer models. Knee.

[28] Iranpour F, Merican AM, Amis AA, Cobb JP (2008). The width:thickness ratio of the patella: an aid in knee arthroplasty. Clin Orthop Relat Res.

[29] Ab Rahman S, Ahmed Shokri A, Ahmad MR, Ismail AF, Termizi NS (2020). Intraoperative patella dimension measurement in asian female patients and its relevance in patellar resurfacing in TKA. Adv Orthop.

[30] Ji G, Wang H, Su X, Wang J, Wang F (2020). The modified semi-tunnel bone bridge technique achieved statistically better knee function than the suture anchor technique. Knee Surg Sports Traumatol Arthrosc.

